# A multicenter, single-arm, basket design, phase II study of NC-6004 plus gemcitabine in patients with advanced unresectable lung, biliary tract, or bladder cancer

**DOI:** 10.18632/oncotarget.27684

**Published:** 2020-08-18

**Authors:** Simona Ruxandra Volovat, Tudor-Eliade Ciuleanu, Piotr Koralewski, Juneko E. Grilley Olson, Adina Croitoru, Krassimir Koynov, Stefano Stabile, Giulio Cerea, Atsushi Osada, Iulian Bobe, Constantin Volovat

**Affiliations:** ^1^Department of Medicine III, Medical Oncology-Radiotherapy, Grigore T. Popa University of Medicine and Pharmacy, Iasi, Romania; ^2^The Oncology Institue "Prof. Dr. Ion Chiricuta" Cluj-Napoca, Cluj-Napoca, Romania; ^3^Iuliu Hatieganu University of Medicine and Pharmacy, Cluj-Napoca, Romania; ^4^Ludwik Rydygier Memorial Specialist Hospital, Krakow, Poland; ^5^UNC Lineberger Comprehensive Cancer Center, Chapel Hill, NC, USA; ^6^Department of Medical Oncology, Fundeni Clinical Institute, Bucharest, Romania; ^7^Multiprofile Hospital for Active Treatment Serdika, Sofia, Bulgaria; ^8^S C Oncologia Falck, Niguarda Cancer Center, Grande Ospedale Metropolitano Niguarda, Milan, Italy; ^9^NanoCarrier Co., Ltd., Tokyo, Japan; ^10^Center of Oncology Euroclinic, Iasi, Romania

**Keywords:** cisplatin, NC-6004, non-small cell lung cancer, biliary tract cancer, bladder cancer

## Abstract

NC-6004 is a nanoparticle developed using micellar technology that can improve release of cisplatin, a standard treatment for many cancer types, and achieve selective distribution to tumors. Here, in the Phase II portion of this study, the activity, safety, tolerability, and effects on quality of life of NC-6004 in combination with gemcitabine was examined in 34 squamous non-small cell lung carcinoma (NSCLC) patients, 50 biliary tract cancer patients, and 13 bladder cancer patients. All patients received 135 mg/m^2^ NC-6004 on day one and 1,250 mg/m^2^ gemcitabine on days one and eight. The median progression-free survival was 3.9 months in NSCLC patients, 4.3 months in biliary tract cancer patients, and 6.8 months in bladder cancer patients fit for cisplatin treatment. The most frequently reported Grade 3 Treatment Emergent Adverse Events across all cohorts were nausea, anemia and neutropenia, and hyponatremia. Quality of life measures for patients who received the combined therapy were generally consistent with expectations for patients undergoing chemotherapy. Overall, combined NC-6004 and gemcitabine treatment resulted in long-lasting antitumor activity and had a favorable safety profile, suggesting that it should be investigated further as a therapy for various types of cancer.

## INTRODUCTION

Platinum-based therapy is widely used to treat many types of cancer. However, cumulative dose-limiting toxicity (DLT) is a common problem with cisplatin treatment [1]. Cisplatin treatment can cause acute myelotoxicity and gastrointestinal toxicity as well as chronic renal-, neuro-, and oto-toxicities after continuous exposure. NC-6004, a nanoparticle that encapsulates cisplatin, was developed to reduce DLTs while maintaining or increasing antitumor activity due to its unique pharmacokinetic characteristics. The NC-6004 nanoparticle is approximately 30 nm in diameter and uses cutting-edge micellar technology; a hydrophilic shell composed of polyethylene glycol increases exposure time in the bloodstream, resulting in increased tumor-specific accumulation [2]. The small size of NC-6004 particles enables increased accumulation and penetration into low permeability organs due to enhanced permeability retention (EPR) compared to liposomal doxorubicin or paclitaxel albumin-bound nanovehicles with larger diameters (90–130 nm) [2, 3]. Every micellar nanoparticle contains an average of 720 cisplatin molecules. The polydispersity index of NC-6004 is 0.070 with a 39% cisplatin content, which is nearly uniformly distributed throughout the micelle. In the presence of chloride in the bloodstream, the NC-6004 micelle formulation is gradually disassembled and releases cisplatin [4]. This sustained release feature of NC-6004 results in a lower maximum cisplatin concentration (C_max_) with a higher area under the curve (AUC); together with preferential distribution to tumors, this reduces toxicity and increases antitumor activity compared to conventional cisplatin at equivalent doses [5]. Stability studies demonstrated that, when 5% dextrose in water is used to reconstitute NC-6004, more than 50% of the cisplatin released from nanoparticles due to the presence of chloride is stable after 120 hours [4].

Fixed concentrations of NC-6004 and increasing concentrations of gemcitabine had synergistic effects in eight human solid tumor cell lines including cisplatin refractory lung, breast, colon, and pancreatic adenocarcinomas. Also, an *in vivo* study demonstrated that tumor size was significantly reduced in human breast, prostate, and lung tumor xenograft models when NC-6004 was combined with gemcitabine compared to treatment with NC-6004 or cisplatin alone. In addition, NC-6004 was better tolerated as indicated by reduced nephrotoxicity and neurotoxicity and showed similar or better antitumor activity compared to cisplatin in preclinical models [6].

In an NC-6004 phase I clinical trial completed in the United Kingdom, administration of NC-6004 in patients with advanced solid tumors was associated with significantly prolonged half-life (230-fold increase) and greater AUC (8.5-fold increase) compared to equivalent cisplatin dose levels. These results indicated that antitumor activity at the maximum tolerated dose (MTD) for NC-6004 may be greater than that of cisplatin, and the NC-6004 dose of 90 mg/m^2^ was determined to be well tolerated [7]. A subsequent phase I/II trial in Asia (NCT02043288) in metastatic pancreatic cancer patients with escalating doses of NC-6004 in combination with gemcitabine and using a traditional 3+3 modified Fibonacci dose escalation design reported a MTD of 120 mg/m^2^ and a Recommended Phase 2 Dose (RP2D) of 90 mg/m^2^ for the combination [8].

In this multicenter phase Ib/II trial (https://clinicaltrials.gov/ Identifier: NCT02240238), we examined NC-6004 in combination with gemcitabine for treating patients with advanced squamous NSCLC, biliary tract, or bladder cancer. The Phase Ib portion of this study was a nonrandomized, open-label, dose escalation and expansion trial using a Bayesian Continual Reassessment Model (N-CRM) and involved 22 patients with solid tumors [13]. The Bayesian model was simulated, designed, and implemented using Fixed and Adaptive Clinical Trial Simulator (FACTS) software version 5.6 [8]. The MTD of NC-6004 was 135 mg/m^2^, which is 50% higher than the MTD determined using a traditional 3+3 modified Fibonacci dose escalation design in a previous trial of NC-6004 that reported no clinically significant neuro, oto, or nephrotoxicity. In the Phase II portion of this study, which is reported here, an adaptive, open-label expansion trial was conducted at the MTD dose identified in the first phase in patients with squamous NSCLC, biliary tract, or bladder cancer; treatment efficacy, safety, and tolerability were assessed [9].

## RESULTS

A total of 97 patients were enrolled in this study between June 2017 and March 2018 at 29 study sites in the United States and Europe. Among them, 34 were diagnosed with squamous NSCLC, 50 with biliary tract carcinoma, and 13 with bladder urothelial cancer. Characteristics of the 97 enrolled patients who received at least one dose of NC-6004 plus gemcitabine are shown in Table 1.

**Table 1:** Demographics of Phase II patients (safety analysis set)

	Cohort 1 (NSCLC)	Cohort 2 (biliary tract cancer)	Cohort 3 (bladder cancer)	Total^a^
	*N* = 33	*N* = 49	*N* = 12	*N* = 97
Age, years, median (range)	61.0 (34–79)	64.0 (35–76)	65.0 (46–80)	63.0 (34–80)
Gender, No. (%)				
Male	26 (78.8)	31 (63.3)	11 (91.7)	70 (72.2)
Female	7 (21.2)	18 (36.7)	1 (8.3)	27 (27.8)
Race, No. (%)				
White	33 (100)	44 (89.8)	11 (91.7)	90 (92.8)
Black	0	1 (2.0)	1 (8.3)	3 (3.1)
Asian	0	2 (4.1)	0	2 (2.1)
Other	0	2 (4.1)	0	2 (2.1)
ECOG at baseline, No. (%)				
Grade 0	2 (6.1)	9 (18.4)	4 (33.3)	16 (16.5)
Grade 1	31 (93.9)	40 (81.6)	8 (66.7)	81 (83.5)
Number of patients with at least one prior chemotherapy	3 (9.1)	3 (6.1)	3 (25.0)	10 (10.3)
Number of patients with at least one prior radiation	11 (33.3)	3 (6.1)	1 (8.3)	15 (15.5)
Number of patients with at least one surgical procedure	12 (36.4)	31 (63.3)	10 (83.3)	56 (57.7)

^a^One patient in each cohort was diagnosed incorrectly, resulting in assignment to the incorrect cohort. These patients are not included in the cohort safety analysis summaries but are included in the total patient number since they received study treatments.

Of the 97 patients initially enrolled, 78 (80.4%) completed the study. Among them, 29 were NSCLC patients (85.3%), 37 were biliary tract cancer patients (74.0%), and 12 were bladder cancer patients (92.3%). The most common reasons for discontinuing treatment were adverse events (AEs), which occurred in 23 patients (23.7%), and disease progression, which occurred in 17 patients (17.5%).

### Efficacy

**Figure 1:**
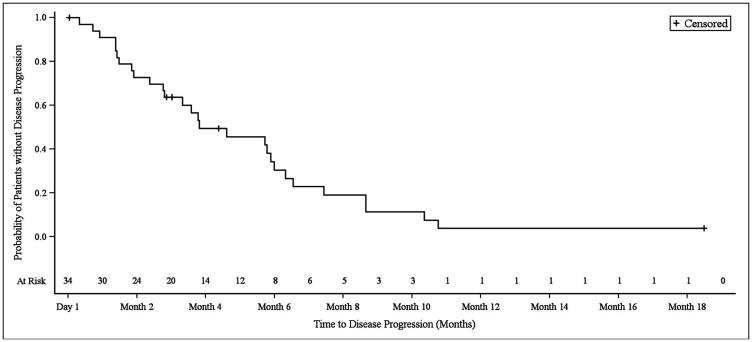
Kaplan–Meier plot of progression-free survival in NSCLC patients.

**Figure 2:**
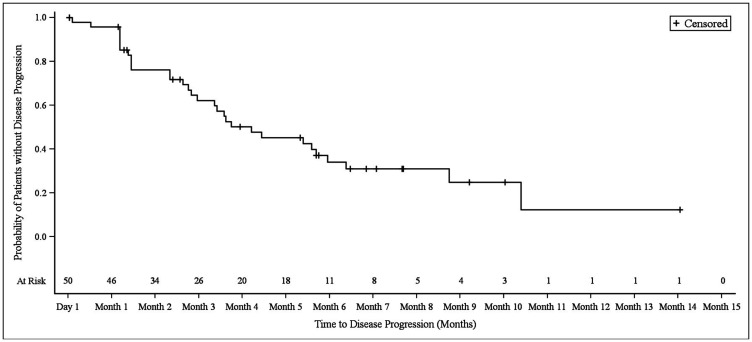
Kaplan–Meier plot of progression-free survival in biliary tract cancer patients.

**Figure 3:**
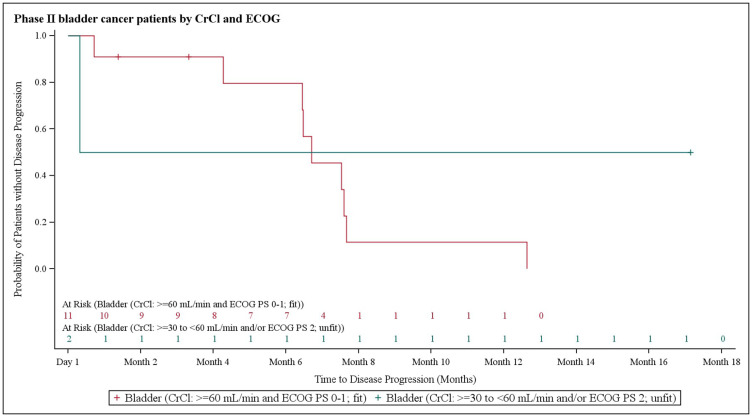
Kaplan–Meier plot of progression-free survival in bladder cancer patients stratified based on creatinine clearance and Eastern Cooperative Oncology Group performance status.

In NSCLC patients, the median progression-free survival (PFS) was 3.9 months (95% CI 2.8–6.1). At the 3.9 month timepoint, 6 NSCLC patients (17.6%) had not shown any disease progression, 19 patients (55.9%) had shown progression, and 9 patients (26.5%) had died (Figure 1). In biliary tract cancer patients, the median PFS was 4.3 months (95% CI 2.9–6.0). At the 4.3 month timepoint, 19 biliary tract cancer patients (38.0%) had not shown disease progression, 22 patients (44.0%) had experienced progression, and 9 patients (18.0%) had died (Figure 2). In bladder cancer patients fit to receive cisplatin treatment, the median PFS was 6.8 months (95% CI 4.3–7.8); PFS could not be evaluated in bladder cancer patients unfit to receive cisplatin. In the overall bladder cancer patient cohort (both fit and unfit for cisplatin) at the 6.8 month timepoint, 3 patients (23.1%) had not shown progression, 8 patients (61.5%) had shown progression, and 2 patients (15.4%) had died (Figure 3). For evaluations of PFS event data, interim analyses were performed every 6 weeks once 10 PFS events were observed. After each interim analysis and at the final analysis, futility was declared in Cohort 1 NSCLC patients and Cohort 2 biliary cancer patients. Due to difficulties enrolling the planned number of patients, recruitment ended prematurely and interim analyses were not performed for Cohort 3 bladder cancer patients.

**Figure 4:**
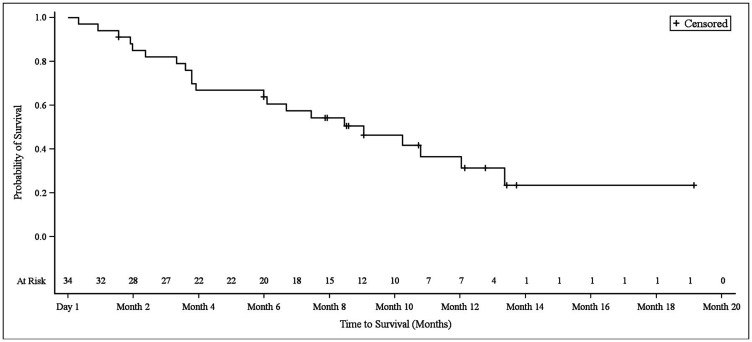
Kaplan–Meier plot of overall survival in NSCLC patients.

**Figure 5:**
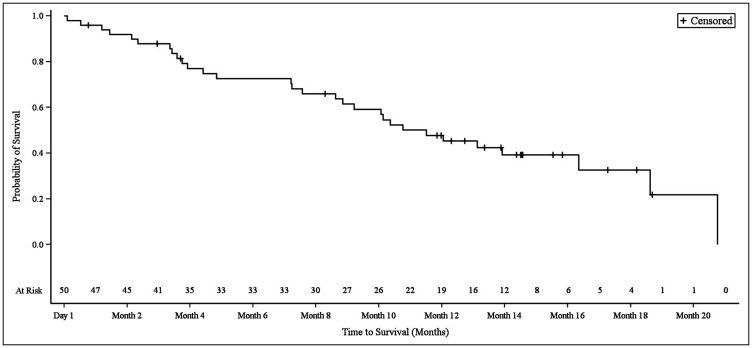
Kaplan–Meier plot of overall survival in biliary tract cancer patients.

**Figure 6:**
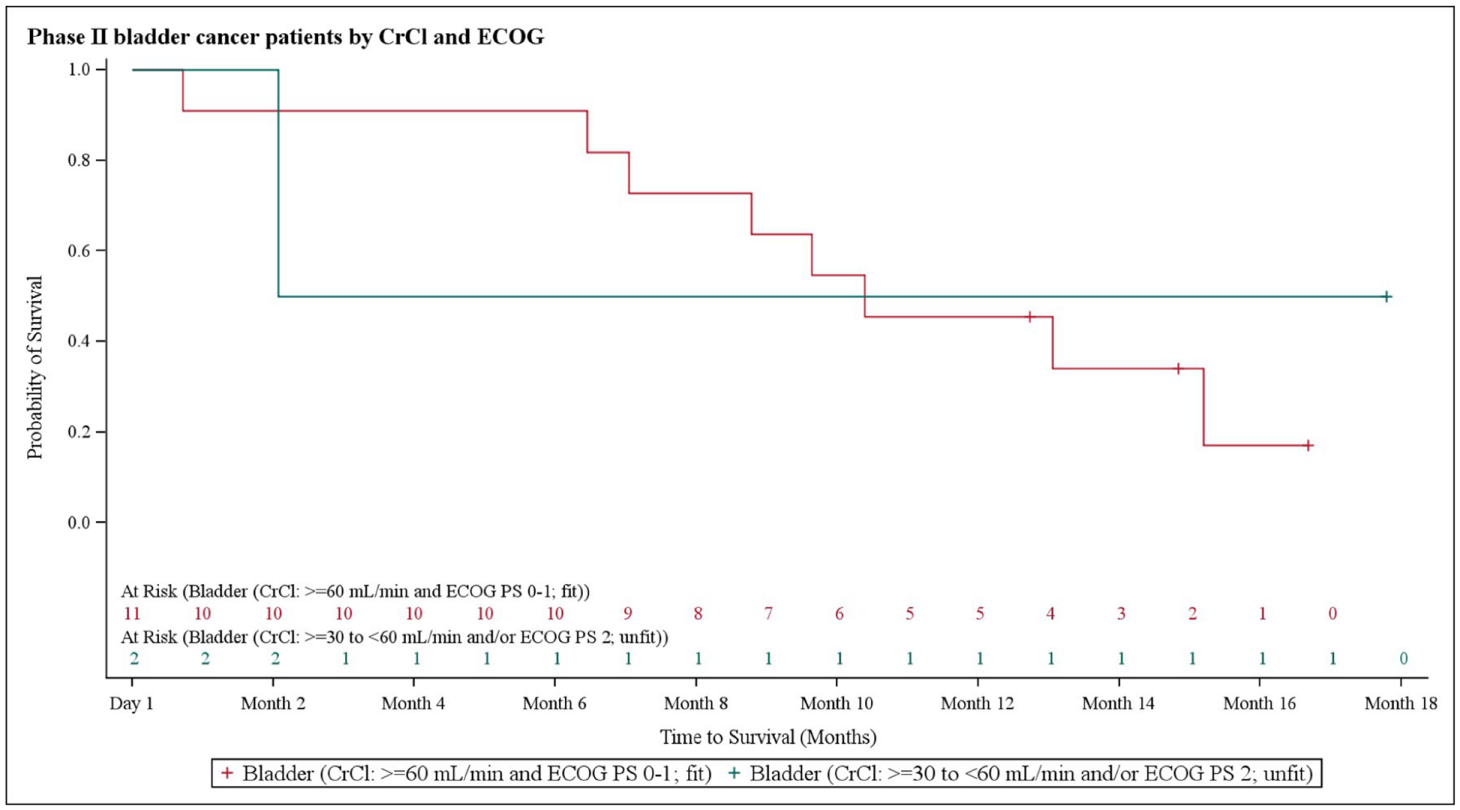
Kaplan–Meier plot of overall survival in bladder cancer patients stratified based on creatinine clearance and Eastern Cooperative Oncology Group performance status.

The median overall survival (OS) in NSCLC patients was 9.2 months (95% CI 4.0–12.2), at which point 13 patients (38.2%) remained alive and 21 patients (61.8%) had died (Figure 4). The median OS in biliary tract cancer patients was 11.7 months (95% CI 8.7–16.6), at which point 20 patients (40.0%) remained alive and 30 patients (60.0%) had died (Figure 5). The median OS in bladder cancer patients was 10.5 months (95% CI 6.5-not applicable), at which point 4 patients (30.8%) remained alive and 9 patients (69.2%) had died (Figure 6).

Duration of response (DOR) was also assessed. In NSCLC patients, at the median DOR of 232.0 days, 1 patient (2.9%) had not shown progression, 3 patients (8.8%) had experienced progression, and 30 patients (88.2%) had not achieved complete response (CR) or partial response (PR). In biliary tract cancer patients, the minimum and maximum DOR values were 92 days and 190 days, respectively (median DOR could not be determined for this cohort). Among cisplatin-fit bladder cancer patients at the median DOR of 133.0 days, 3 patients (23.1%) had experienced progression and 10 patients (76.9%) had not achieved CR or PR.

Disease control rates were evaluated by determining the proportion of patients who showed the best overall response of stable disease (SD) for longer than 7 weeks and for whom PR or CR was confirmed when the patient discontinued study treatment. Successful disease control was observed in 12 NSCLC patients (35.3%), 19 biliary tract cancer patients (38.0%), and 7 bladder cancer patients (53.8%) based on these criteria.

### Safety

Treatment Emergent Adverse Events (TEAEs) that were observed in at least 5% of all patients are shown in Table 2. Most TEAEs were Grade 1 or Grade 2 in intensity in Part 2 as well. Overall, there were 8 Grade 5 TEAEs, 22 Grade 4 TEAEs, and 250 Grade 3 TEAEs during the course of the study. One patient experienced cardiac arrest and another patient experienced pulmonary artery thrombosis; all other Grade 5 TEAEs were considered unrelated to the medications administered in this study. The only Grade 4 TEAEs experienced by more than one patient in any cohort were increased gamma-glutamyl transpeptidase (GGT) (12.2%), neutropenia (6.1%), and decreased platelet count (4.1%), and all of these events occurred in biliary tract cancer patients.

**Table 2:** Treatment Emergent Adverse Events occurring in ≥ 5% of all patients

	*N* = 34	*N* = 49	*N* = 13	*N* = 97^a^
	*n* (%)	*n* (%)	*n* (%)	*n* (%)
Total number of TEAEs	334	1003	172	1541
Number of patients with at least one TEAE	33 (100.0)	49 (100.0)	12 (100.0)	97 (100.0)
Nausea	15 (45.5)	31 (63.3)	8 (66.7)	56 (57.7)
Hypomagnesaemia	16 (48.5)	23 (46.9)	5 (41.7)	47 (48.5)
Anemia	16 (48.5)	18 (36.7)	4 (33.3)	38 (39.2)
Vomiting	7 (21.2)	23 (46.9)	4 (33.3)	35 (36.1)
Blood creatinine increased	6 (18.2)	12 (24.5)	5 (41.7)	24 (24.7)
Neutropenia	11 (33.3)	11 (22.4)	2 (16.7)	24 (24.7)
Alanine aminotransferase increased	6 (18.2)	15 (30.6)	1 (8.3)	22 (22.7)
Decreased appetite	7 (21.2)	14 (28.6)	1 (8.3)	22 (22.7)
Aspartate aminotransferase increased	5 (15.2)	15 (30.6)	1 (8.3)	21 (21.6)
Fatigue	3 (9.1)	16 (32.7)	2 (16.7)	21 (21.6)
Thrombocytopenia	5 (15.2)	13 (26.5)	1 (8.3)	19 (19.6)
Constipation	4 (12.1)	13 (26.5)	1 (8.3)	18 (18.6)
Hypokalemia	5 (15.2)	8 (16.3)	4 (33.3)	17 (17.5)
Asthenia	8 (24.2)	8 (16.3)	0	16 (16.5)
Gamma-glutamyl transferase increased	0	15 (30.6)	1 (8.3)	16 (16.5)
Hyponatremia	7 (21.2)	6 (12.2)	1 (8.3)	14 (14.4)
Insomnia	4 (12.1)	8 (16.3)	2 (16.7)	14 (14.4)
Pyrexia	1 (3.0)	10 (20.4)	2 (16.7)	13 (13.4)
Diarrhea	1 (3.0)	7 (14.3)	1 (8.3)	10 (10.3)
Platelet count decreased	1 (3.0)	8 (16.3)	1 (8.3)	10 (10.3)
Back pain	1 (3.0)	6 (12.2)	2 (16.7)	9 (9.3)
Dysgeusia	0	7 (14.3)	1 (8.3)	9 (9.3)
Paresthesia	2 (6.1)	7 (14.3)	0	9 (9.3)
Blood alkaline phosphatase increased	0	7 (14.3)	1 (8.3)	8 (8.2)
Blood bilirubin increased	0	8 (16.3)	0	8 (8.2)
Neutrophil count decreased	3 (9.1)	3 (6.1)	1 (8.3)	8 (8.2)
Edema peripheral	2 (6.1)	4 (8.2)	1 (8.3)	8 (8.2)
Weight decreased	3 (9.1)	3 (6.1)	2 (16.7)	8 (8.2)
Abdominal pain	0	7 (14.3)	0	7 (7.2)
Abdominal pain	0	5 (10.2)	1 (8.3)	6 (6.2)
Dehydration	1 (3.0)	4 (8.2)	1 (8.3)	6 (6.2)
Dyspnea	4 (12.1)	2 (4.1)	0	6 (6.2)
Headache	1 (3.0)	5 (10.2)	0	6 (6.2)
Hiccups	0	5 (10.2)	1 (8.3)	6 (6.2)
Arthralgia	1 (3.0)	2 (4.1)	1 (8.3)	5 (5.2)
Dizziness	2 (6.1)	2 (4.1)	0	5 (5.2)
Non-cardiac chest pain	1 (3.0)	4 (8.2)	0	5 (5.2)
Weight increased	1 (3.0)	2 (4.1)	2 (16.7)	5 (5.2)

One patient in each cohort was diagnosed incorrectly and was therefore assigned to the incorrect cohort. These patients are not included in the safety analyses but are included in the total patient number since they did receive drug treatments as part of this study.

The most frequently reported Grade 3 TEAEs were: anemia and neutropenia (27.3% each) and hyponatremia (18.2%) in NSCLC patients; nausea, neutropenia, and thrombocytopenia (14.3% each) in biliary tract cancer patients; and anemia (25.0%), neutropenia, hypokalemia, hypomagnesemia, and hypertension (16.7% each) in bladder cancer patients. Grade 3 hypokalemia, hypomagnesemia, anemia, and neutropenia were reported in at least 2 patients in each cohort.

### Quality of life

Patients’ quality of life was assessed by determining whether EORTC QLQ-C30 remained stable or decreased with regard to functional scales and remained stable or increased with regard to symptoms scales, as would be expected in patients who are receiving chemotherapy treatment. By the end of treatment, one patient’s ECOG performance status scores had worsened; this patient shifted from Grade 0 to Grade 1.

### Pharmacokinetics

From the intensive sampling in Part 1, the major pharmacokinetic parameters were estimated based on total platinum concentrations. As shown in Table 3, total plasma clearance (CL) was 65.0 ± 15.6 mL/hr, steady state volume of distribution (V_ss_) was 3.9 ± 1.7 L, and elimination half-life (T_1/2_) was 91.2 ± 19.5 hr.

**Table 3:** Major plasma pharmacokinetic parameters and platinum concentrations at specific time points for NC-6004 at 135 mg/m^2^

		Total Platinum^a^		Free Platinum^b^	
		Mean	(S. D.)	Mean	(S. D.)
C_max_	μg/mL	50.08	(9.84)	NA	NA
C_1 hr_	μg/mL	48.82	(11.09)	0.192	(0.164)
C_168 hr_	μg/mL	1.20	(0.42)	0.057	(0.119)
C_trough_	μg/mL	0.44	(0.14)	0.031	(0.077)
AUC_0-inf_	μg^*^hr/mL	2611.0	(520.7)	NA	NA
CL	mL/hr	65.0	(15.6)	NA	NA
V_ss_	L	3.9	(1.7)	NA	NA
T_1/2_	Hr	91.2	(19.5)	NA	NA

^a^Data from Part 1; ^b^Data from Part 2.

**Figure 7:**
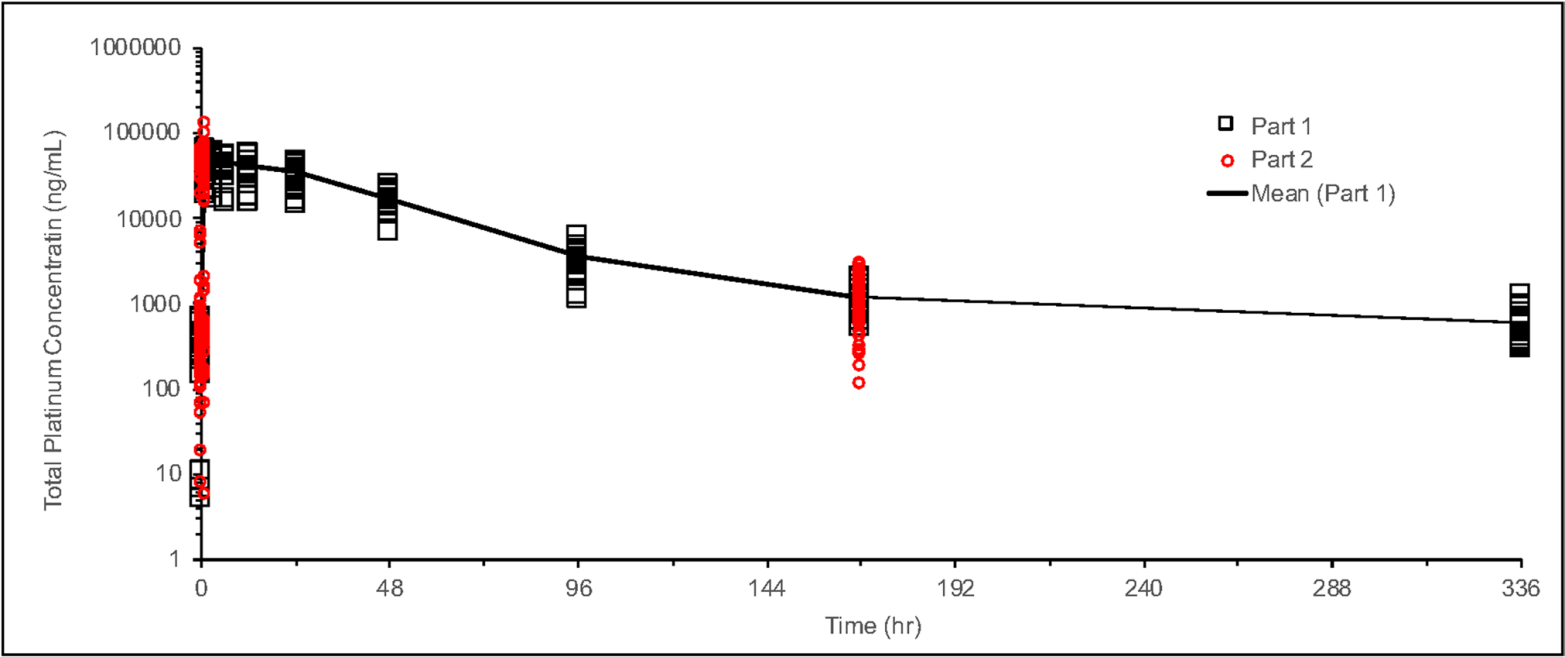
Time profile of total platinum concentration for NC-6004 at 135 mg/m^2^. Phase Ib data are shown as black circles and Phase II data as red circles.

**Figure 8:**
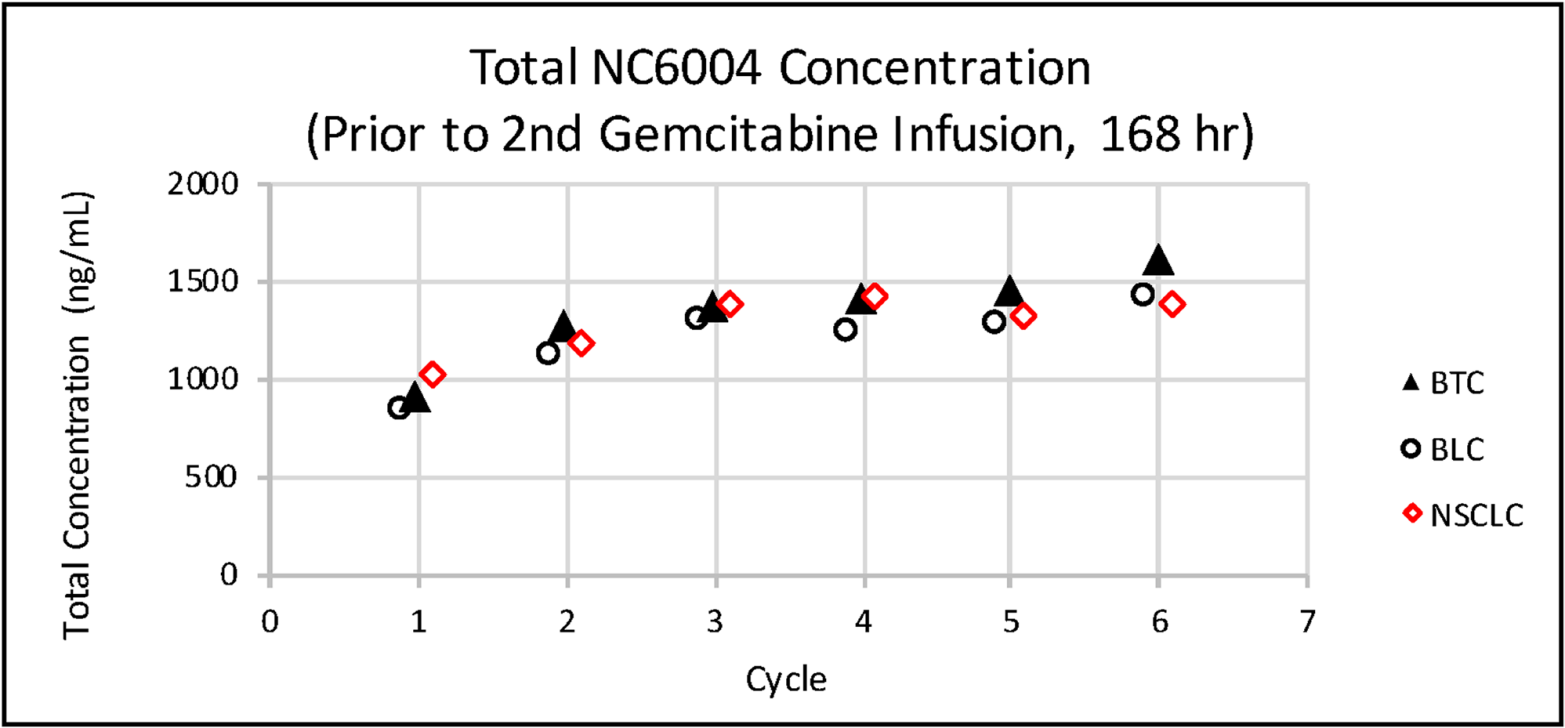
Mean total platinum concentrations prior to the second gemcitabine infusion (168 h) in each cycle in patients with various tumor types.

**Figure 9:**
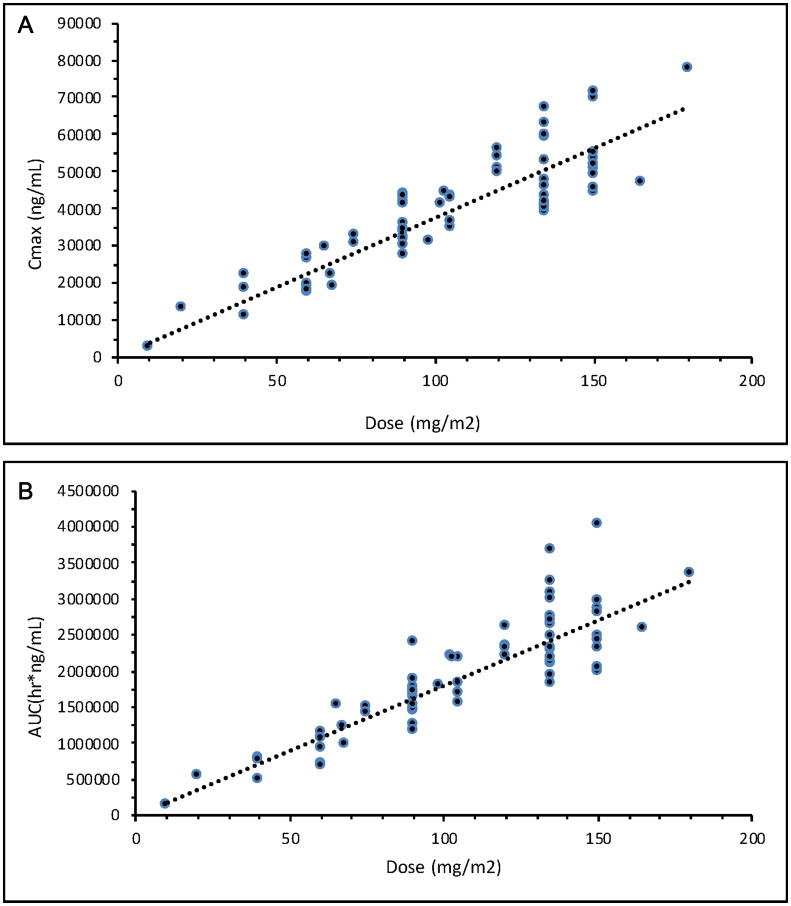
(**A**) Total cisplatin exposure as indicated by C_max_ after NC-6004 doses ranging from 10–180 mg/m^2^. (**B**) Total cisplatin exposure as indicated by AUC after NC-6004 doses ranging from 10–180 mg/m^2^.

Data collected from sparse plasma samples in Phase II of this study were compared to the full time-dependent concentration profile based on data from intensive plasma samples from the 135 mg/m^2^ dose group in the previous Phase Ib portion of the study. As shown in Figure 7, platinum concentrations at the pre-dose (0 hr), end of NC-6004 infusion (1 hr), and start of second gemcitabine infusion (168 hr) timepoints in Phase II were consistent with those in Phase Ib. Levels were also evaluated at various other time points and analyzed in multiple cycles across the tumor types. As shown in Figure 8, there was no platinum accumulation from cycle to cycle, nor were there any differences among patients with different tumor types. NC-6004 has been evaluated in several studies at doses ranging from 10–180 mg/m^2^; C_max_ and AUC values from these studies increased with dose in a linear fashion as shown in Figure 9A and 9B.

## DISCUSSION

In this histology-independent, early phase II basket study of combination treatment with NC-6004 and gemcitabine, we observed a median PFS of 3.9 months for advanced squamous cell NSCLC. This is shorter than the median PFS of 5.1 months observed previously in a Phase III study of cisplatin-gemcitabine [10, 11]. In those studies, the median OS was 9.2 months in patients who received a NC-6004-gemcitabine combination, while median OS values of 8.9 months and 10.3 months were observed in patients who received the cisplatin-gemcitabine combination [10, 11].

In this study, we observed a median PFS of 4.3 months in biliary tract cancer patients who received the NC-6004-gemcitabine combination. In a previous phase III ABC-02 study, a median PFS of 8.0 months was observed in biliary tract cancer patients treated with a cisplatin-gemcitabine combination; in the same study, the median OS was 11.7 months both in patients treated with NC-6004-gemcitabine and in those treated with cisplatin-gemcitabine [12].

Finally, we observed a median PFS of 6.8 months in cisplatin-fit bladder cancer patients receiving a NC-6004-gemcitabine combination. A previous phase III clinical trial found a median PFS of 7.4 months in bladder cancer patients receiving cisplatin-gemcitabine; in the same study, median OS was 10.5 months for patients treated with NC-6004-gemcitabine and 13.8 months in patients who received cisplatin-gemcitabine [13].

Pocock raised important concerns regarding the use of historical control [10]. Such limitations preclude our ability to conclusively determine the antitumor activity of combined NC-6004-gemcitabine treatments based on previous studies. Differences in patient backgrounds and demographics, among other factors, prevented us from comparing the efficacy, safety, tolerability, QoL impact, and pharmacokinetics of our combined NC-6004-gemcitabine treatment against historical data on combined cisplatin-gemcitabine treatments in this study. As expected, our attempts to compare outcomes associated with conventional cisplatin regimens in previous studies to our combined treatment were inconclusive; future randomized studies would be needed to directly compare these treatments.

Here, combined NC-6004-gemcitabine therapy was well tolerated, as was the case in a Phase I/II study performed in Asia in pancreatic cancer patients (Study NC-6004-002) that demonstrated a toxicity profile similar to cisplatin [14]. Most of the AEs observed in this study were Grade 1 or Grade 2 in intensity, and the most common TEAEs related to study treatment were nausea, hypomagnesemia, and anemia; this suggests that the NC-6004-gemcitabine combination may be safer than cisplatin-gemcitabine.

Toxicities associated with cisplatin chemotherapy and gastrointestinal and hematological toxicities associated with gemcitabine are well-known. However, our QoL data suggest that use of the NC-6004 nanoparticle vehicle benefitted patients by reducing treatment toxicity. Additional studies including a cisplatin-gemcitabine treatment group for direct comparison are needed to conclusively demonstrate such a benefit.

The values for pharmacokinetic parameters observed in this study are consistent with previously reported ranges [7]. However, compared to conventional cisplatin similarly administered in a one-hour infusion [15], the total platinum delivered through NC-6004 has a significantly lower CL and much smaller V_ss_, leading to a longer T_1/2_. Total and free platinum concentrations at different time points are listed in Table 3. The free platinum in the ultrafiltrate assayed in this study represents the fraction of cisplatin released from NC-6004 micelles that is not bound to plasma proteins. The free platinum concentration at the end of the one-hour infusion of 135 mg/m^2^ NC-6004, which was similar to the C_max_, was 0.19 ± 0.16 μg/mL (Table 3). This is markedly lower than the free platinum C_max_ of 4.3 ± 0.7 μg/mL achieved by a one hour infusion of conventional cisplatin at the lower dose of 100 mg/m^2^ [16]. However, the free platinum concentration of 0.057 ± 0.12 μg/mL measured at 168 hr (Day 7) indicates that its elimination was significantly slower, and exposure duration was prolonged, after release from NC-6004 micelles. In addition, the release of cisplatin from NC-6004 appears to be time- and concentration-dependent; the free/total platinum concentration ratio was only 0.4% at 1 hr, but increased to 4.8% at 168 hr and 7.1% at the trough immediately prior to the NC-6004 dose in the subsequent cycle. Single gemcitabine infusions were administered on days one and eight of each treatment cycle in this study. Total plasma CL and V_ss_ values for NC-6004 in the presence of gemcitabine were similar to those observed in previous studies where NC-6004 was administered either alone or in combination with other agents.

This study supports nonclinical findings that slow release of cisplatin from NC-6004 resulted in long-lasting systemic exposure to cisplatin, and thereby prolonging its antitumor effects, with a toxicity profile similar to that of cisplatin. In addition, combined treatment with NC-6004 and gemcitabine was well tolerated, with a manageable toxicity profile similar to cisplatin. After combined treatment, QoL as assessed by EORTC QLQ-C30 either remained the same or decreased as expected for patients receiving chemotherapy.

Accumulation index values of 0.60 to 1.21 across all dose levels indicated that there was little accumulation of total platinum in the plasma following a single IV administration of NC-6004 in 3-week treatment cycles. Finally, our survival analysis demonstrated that the NC-6004-gemcitabine combination is a safe and effective option for front-line treatment of advanced cancers and yields comparable results to combined cisplatin-gemcitabine treatment.

## MATERIALS AND METHODS

This study covers the Phase II expansion trial portion of a multicenter, single-arm, basket design study (https://clinicaltrials.gov/ Identifier: NCT02240238). In Phase Ib, the MTD of NC-6004 in combination with gemcitabine was determined using a Bayesian model in patients with solid tumors.

### Patient population

Eligibility criteria were as follows: histologically or cytologically confirmed diagnosis of stage IV squamous NSCLC, advanced or metastatic biliary tract carcinoma (intrahepatic or extrahepatic cholangiocarcinoma, gallbladder cancer, or ampullary carcinoma), or stage IV bladder carcinoma; no prior systemic anticancer therapy for advanced or metastatic disease; measurable disease per Response Evaluation Criteria in Solid Tumors Version 1.1 (RECIST); Eastern Cooperative Oncology Group (ECOG) performance status (PS) of 0 to 1 in NSCLC and biliary tract cancer cohorts and 0 to 2 in bladder carcinoma cohort; adequate bone marrow reserve (absolute neutrophil count ≥ 1.5 × 10^9^/L, platelet count ≥ 100 × 10^9^/L, and hemoglobin level ≥ 10 g/dL); acceptable organ function criteria (total serum bilirubin < 1.5 × upper limit of normal [ULN], baseline alanine transaminase, aspartate transaminase < 2 × ULN or in patients with documented hepatic metastases < 5.0 × ULN, serum creatinine < 1.5 mg/dL). Bladder cancer patients were stratified by creatinine clearance (CrCl,) ≥ 30 to < 60 mL/min and/or ECOG PS 2 (cisplatin-unfit population) and CrCl ≥ 60 mL/min and ECOG PS 0 to 1 (cisplatin-fit population). The patients were separated into 3 cohorts based on cancer type: squamous NSCLC, biliary tract carcinoma, and bladder carcinoma.

The study was conducted in compliance with the Declaration of Helsinki, International Conference on Harmonization Guidelines for Good Clinical Practice, and applicable local regulations.

### Trial design

The primary objective of this trial was to evaluate the activity of NC-6004 in combination with gemcitabine in patients with first-line Stage IV squamous NSCLC, advanced or metastatic biliary tract cancer, and metastatic or locally advanced bladder cancer. Efficacy was evaluated based on response rates at week 8 as assessed by the site investigators according to RECIST, version 1.1.

The secondary objectives were to evaluate the safety, quality of life, pharmacokinetics, and antitumor activity (Objective Response Rate [ORR], Disease Control Rate [DCR], Duration of Response [DOR], Progression-Free Survival [PFS], and Overall Survival [OS]) of NC-6004 when combined with gemcitabine.

PFS was the primary endpoint in this study and was continuously updated within each cohort and compared to historical PFS from pivotal Phase III cisplatin and gemcitabine studies. The PFS hazard model was updated as PFS data accrued, and PFS hazard ratios (HR) were calculated for each cohort (Table 4).

**Table 4:** Historical controls for progression-free survival for each cohort

Cohort	Biliary Tract Cancer	Bladder Cancer (CrCl: ≥ 60 mL/min and ECOG PS 0,1; fit)	Bladder Cancer (CrCl: ≥ 30 to < 60 mL/min and/or ECOG PS 2; unfit)	Squamous NCSLC
Historical median PFS duration	8.8 months	7.6 months	5.8 months	5 months

Abbreviations: CrCl, creatinine clearance; ECOG, Eastern Cooperative Oncology Group; NSCLC, non-small cell lung cancer; PFS, progression-free survival; PS, performance status.

NC-6004 was administered in a 1-hour intravenous infusion on day 1 of each 21-day cycle. All patients received pre- (minimum of 1 L over 1–3 hours) and post-infusion (minimum of 500 mL over 2 hours) hydration regimens of NC- 6004 with 0.9% sodium chloride (0.45% sodium chloride was allowed at the investigator’s discretion). To reduce the risk of hypersensitivity reactions, all patients received an antihistamine (diphenhydramine 50 mg, ranitidine 50 mg, or 20 mg famotidine) intravenously 30 minutes prior to administration of NC-6004 as well as 20 mg dexamethasone orally 12 and 6 hours prior to and 4 mg twice daily for two days after NC-6004 infusion. The use of antiemetic agents was allowed based on standard treatment center protocols for cisplatin-based regimens but was not mandated. NC-6004 was administered at a dose of 135 mg/m^2^ in a 1-hour intravenous infusion on day 1 of each cycle, and gemcitabine was administered at 1,250 mg/m^2^ in a 30 minute-intravenous infusion following NC-6004 infusion on day 1 and on day 8 of each cycle. All patients were treated until they experienced disease progression, unacceptable toxicity, or withdrew from the trial, whichever occurred first.

Adverse events related to treatment were graded using the National Cancer Institute Common Terminology Criteria for Adverse Events Version 4.03 (NCI CTCAE). QoL was assessed using the European Organization for Research and Treatment of Cancer Quality of Life Questionnaire-Core 30 (EORTC QLQ-C30). Pharmacokinetic samples were collected, total platinum (including cisplatin both encapsulated in and released from the NC-6004 micelle formulation) and released platinum concentrations in the plasma were measured, and key pharmacokinetic parameters were determined.

The patients were separated into the following three cohorts based on cancer type: Cohort 1: first-line metastatic squamous NSCLC; Cohort 2: first-line metastatic or locally advanced cholangiocarcinoma, gallbladder cancer, or ampullary cancer (biliary tract cancer); and Cohort 3: first-line metastatic or locally advanced transitional cell carcinoma of the urinary tract (bladder cancer). Bladder cancer patients in Cohort 3 were stratified according to creatinine clearance (CrCl) rates to assess study treatment in patients with reduced kidney function (CrCl: ≥ 30 to < 60 mL/min [cisplatin unfit] and ≥ 60 mL/min [cisplatin fit]) in a controlled manner with the stipulation that enrolment would stop if CrCl rates decreased by more than 50% from baseline in 2 consecutive assessments at least one week apart in 2 of 6 patients in the cisplatin unfit group.

The initial plan involved enrolling up to 50 patients each in Cohorts 1 and 2 and up to 60 patients (i.e., 30 unfit and 30 fit bladder cancer patients) in Cohort 3, for a total of up to 160 patients. Patients were slated to receive up to 6 cycles of treatment for Cohorts 1 and 3 or 8 cycles for Cohort 2.

Sparse plasma samples were collected immediately before (0 hr) and after NC-6004 infusion (1 hr) and prior to the second gemcitabine infusion (168 hr) in each cycle from all patients in the Phase II portion of this study for up to 8 cycles. Concentrations of total platinum and platinum remaining after ultrafiltration to eliminate free platinum were measured in all samples. In the Phase Ib portion of this study, in which the MTD of 135 mg/m^2^ was identified, intensive plasma samples were taken for PK profiling at 0, 1, 3, 6, 12, 24, 48, 96, 168, and 336 hours from the start of NC-6004 infusion from all subjects in Cycles 1, 3, and 5. Only total platinum concentrations were measured in these samples; free platinum concentrations could not be measured due a technical issue. Concentration data were imported into Phoenix WinNonlin (v8.2, Certara, Princeton, NJ, USA) for PK parameter calculations in a non-compartmental model.

### Statistical analysis

PFS was continuously updated and compared to historical PFS values from pivotal Phase 3 cisplatin and gemcitabine studies for patients in each cohort. The PFS hazard model was updated as PFS data accrued, and a PFS hazard ratio (HR) compared to the historical control was obtained for each cohort. Once 10 PFS events were observed, interim analyses were performed every 6 weeks. At each interim analysis and the final analysis, there were 3 possible outcomes for each cohort: 1. Futility-10 PFS events were observed in each cohort and at least 1 of the following was true: Probability (Promising-HR less than 0.85.) < 0.4; Probability (Phase 3 Success-HR of 0.75) < 0.4; 2. Success-25 PFS events were observed in each 50-patient cohort and 15 PFS events in each 30-patient bladder cancer cohort, and Probability (Phase 3 Success) > 0.8; 3. Inconclusive, neither futility nor success.

Safety and efficacy analysis were conducted for patients who received at least one drug dose as part of this study. PFS and OS were estimated using the Kaplan–Meier method. All analyses were conducted using SAS^®^ software Version 9.3 (SAS Institute, Inc., Cary, NC, USA).

## Abbreviations

AE: Adverse Event
AUC: Area Under the Curve
BTC: Biliary Tract Cancer
BLC: Bladder Cancer
CL: Clearance
C_max_: Maximum concentration
CR: Complete Response
CrCl: Creatinine Clearance
CTCAE: Common Terminology Criteria for Adverse Events
DCR: Disease Control Rate
DLT: Dose Limiting Toxicity
DOR: Duration of Response
ECOG: Eastern Cooperative Oncology Group
EORTC QLQ-C30: European Organisation for Research and Treatment of Cancer Quality of Life Questionnaire-Core 30
EPR: Enhanced Permeability and Retention
FACTS: Fixed and Adaptive Clinical Trial Simulator
GGT: Gamma-Glutamyl Transpeptidase
HR: Hazard Ratio
MTD: Maximum Tolerated Dose
NCI: National Cancer Institute
NSCLC: Non-Small Cell Lung Cancer
ORR: Overall Response Rate
OS: Overall Survival
PFS: Progression Free Survival
PK: Pharmacokinetic
PR: Partial Response
PS: Performance Status
QoL: Quality of Life
RECIST: Response Evaluation Criteria in Solid Tumors
RP2D: Recommended Phase 2 Dose
SD: Stable Disease
S.D: Standard Deviation
T_1/2_: Elimination half-life
TEAE: Treatment Emergent Adverse Event
ULN: Upper Limit of Normal
Vss: Volume of distribution at steady state

## References

[1] Andersson A , Fagerberg J , Lewensohn R , Ehrsson H . Pharmacokinetics of Cisplatin and Its Monohydrated Complex in Humans. J Pharm Sci. 1996; 85:824–827. 10.1021/js960037a. 8863271

[2] Baba M , Matsumoto Y , Kashio A , Cabral H , Nishiyama N , Kataoka K , Yamasoba T . Micellization of cisplatin (NC-6004) reduces its ototoxicity in guinea pigs. J Control Release. 2012; 157:112–117. 10.1016/j.jconrel.2011.07.026. 21807044

[3] Cabral H , Matsumoto Y , Mizuno K , Chen Q , Murakami M , Kimura M , Terada Y , Kano MR , Miyazono K , Uesaka M , Nishiyama N , Kataoka K . Accumulation of sub-100 nm polymeric micelles in poorly permeable tumours depends on size. Nat Nanotechnol. 2011; 6:815–823. 10.1038/nnano.2011.166. 22020122

[4] Hartmann JT , Lipp HP . Toxicity of platinum compounds. Expert Opin Pharmacother. 2003; 4:889–901. 10.1517/14656566.4.6.889. 12783586

[5] Korst AE , van der Sterre ML , Gall HE , Fichtinger-Schepman AM , Vermorken JB , van der Vijgh WJ . Influence of Amifostine on the Pharmacokinetics of Cisplatin in Cancer Patients. Clin Cancer Res. 1998; 4:331–336. 9516919

[6] Matsumura Y , Maeda H . A new concept for macromolecular therapeutics in cancer chemotherapy: mechanism of tumoritropic accumulation of proteins and the antitumor agent smancs. Cancer Res. 1986; 46:6387–6392. 2946403

[7] Nishiyama N , Okazaki S , Cabral H , Miyamoto M , Kato Y , Sugiyama Y , Nishio K , Matsumura Y , Kataoka K . Novel cisplatin-incorporated polymeric micelles can eradicate solid tumors in mice. Cancer Res. 2003; 63:8977–8983. 14695216

[8] Plummer R , Wilson RH , Calvert H , Boddy AV , Griffin M , Sludden J , Tilby MJ , Eatock M , Pearson DG , Ottley CJ , Matsumura Y , Kataoka K , Nishiya T . A phase I clinical study of cisplatin-incorporated polymeric micelles (NC- 6004) in patients with solid tumours. Br J Cancer. 2011; 104:593–598. 10.1038/bjc.2011.6. 21285987 PMC3049602

[9] Subbiah V , Grilley-Olson JE , Combest AJ , Sharma N , Tran RH , Bobe I , Osada A , Takahashi K , Balkissoon J , Camp A , Masada A , Reitsma DJ , Bazhenova LA . Phase Ib/II Trial of NC-6004 (Nanoparticle Cisplatin) Plus Gemcitabine in Patients with Advanced Solid Tumors. Clin Cancer Res. 2018; 24:43–51. 10.1158/1078-0432.CCR-17-1114. 29030354

[10] Pocock SJ . The combination of randomized and historical controls in clinical trials. J Chronic Dis. 1976; 29:175–188. 10.1016/0021-9681(76)90044-8. 770493

[11] Scagliotti GV , Parikh P , von Pawel J , Biesma B , Vansteenkiste J , Manegold C , Serwatowski P , Gatzemeier U , Digumarti R , Zukin M , Lee JS , Mellemgaard A , Park K . Phase III Study Comparing Cisplatin Plus Gemcitabine With Cisplatin Plus Pemetrexed in Chemotherapy-Naïve Patients With Advanced-Stage Non–Small-Cell Lung Cancer. J Clin Oncol. 2008; 26:3543–3551. 10.1200/JCO.2007.15.0375. 18506025

[12] Smit EF , van Meerbeeck JP , Lianes P , Debruyne C , Legrand C , Schramel F , Smit H , Gaafar R , Biesma B , Manegold C , Neymark N , Giaccone G , and European Organization for Research and Treatment of Cancer Lung Cancer Group. Three-arm randomized study of two cisplatin-based regimens and paclitaxel plus gemcitabine in advanced non-small-cell lung cancer: a phase III trial of the European Organization for Research and Treatment of Cancer Lung Cancer Group–EORTC 08975. J Clin Oncol. 2003; 21:3909–3917. 10.1200/JCO.2003.03.195. 14581415

[13] Socinski MA , Schell MJ , Peterman A , Bakri K , Yates S , Gitten R , Unger P , Lee J , Lee JH , Tynan M , Moore M , Kies MS . Phase III trial comparing a defined duration of therapy versus continuous therapy followed by second-line therapy in advanced- Stage IIIB/IV non–small-cell lung cancer. J Clin Oncol. 2002; 20:1335–1343. 10.1200/jco.2002.20.5.1335. 11870177

[14] Uchino H , Matsumura Y , Negishi T , Koizumi F , Hayashi T , Honda T , Nishiyama N , Kataoka K , Naito S , Kakizoe T . Cisplatin-incorporating polymeric micelles (NC 6004) can reduce nephrotoxicity and neurotoxicity of cisplatin in rats. Br J Cancer. 2005; 93:678–687. 10.1038/sj.bjc.6602772. 16222314 PMC2361620

[15] Valle J , Wasan H , Palmer DH , Cunningham D , Anthoney A , Maraveyas A , Madhusudan S , Iveson T , Hughes S , Pereira SP , Roughton M , Bridgewater J , and ABC-02 Trial Investigators. Cisplatin plus Gemcitabine versus Gemcitabine for Biliary Tract Cancer. N Engl J Med. 2010; 362:1273–1281. 10.1056/NEJMoa0908721. 20375404

[16] von der Maase H , Hansen SW , Roberts JT , Dogliotti L , Oliver T , Moore MJ , Bodrogi I , Albers P , Knuth A , Lippert CM , Kerbrat P , Sanchez Rovira P , Wersall P , et al. Gemcitabine and cisplatin versus methotrexate, vinblastine, doxorubicin, and cisplatin in advanced or metastatic bladder cancer: results of a large, randomized, multinational, multicenter, phase III study. J Clin Oncol. 2000; 18:3068–77. 10.1200/JCO.2000.18.17.3068. 11001674

